# Prevalence of metabolic syndrome in the Brazilian Xavante indigenous population

**DOI:** 10.1186/s13098-015-0100-x

**Published:** 2015-11-21

**Authors:** Luana Padua Soares, Amaury Lelis Dal Fabbro, Anderson Soares Silva, Daniela Saes Sartorelli, Luciana Ferreira Franco, Patrícia Chamadoira Kuhn, Regina Santiago Moises, João Paulo Botelho Vieira-Filho, Laércio Joel Franco

**Affiliations:** Medical School, Federal University of Uberlândia, Av. Pará 1720, Bloco 2U, Uberlândia, MG CEP 38405-320 Brazil; Department of Social Medicine, Ribeirão Preto Medical School, University of São Paulo, Av. Bandeirantes, 3900, Ribeirão Preto, SP CEP 14049-900 Brazil; Division of Endocrinology, Escola Paulista de Medicina, Federal University of São Paulo, Rua Pedro de Toledo, 781/12º andar, São Paulo, SP CEP 04039-001 Brazil

**Keywords:** Metabolic syndrome, Indian population, Weight excess, Obesity

## Abstract

**Background:**

The raising prevalence of weight excess and of non-communicable diseases in indigenous populations, as well as changes in food consumption and reduction in the frequency and intensity of physical activity, suggest that the prevalence of metabolic syndrome (MS) is also elevated. The objective of this study was to evaluate the prevalence of MS and the frequency of its components in the Xavante adult population living in the Indian reservations of São Marcos and Sangradouro/Volta Grande, in the state of Mato Grosso, Brazil. A cross-sectional study was carried out among 932 Xavante Indians aged 20 years or more, in the 2008–2012 period. The variables analysed were gender, age, weight, height, waist circumference, blood pressure, initial and 2-h capillary glycemia in a 75 g OGTT, levels of triglycerides and HDL-cholesterol. The diagnostic criteria for MS proposed by the IDF and AHA/NHLBI were used.

**Results:**

The prevalence of MS was 66.1 % (95 % CI 63.0–69.2), being 76.2 % (95 % CI 72.4–80.0) in women and 55.6 % (95 % CI 51.0–60.2) in men. Women had higher prevalence of MS in all age groups. Elevated waist circumference and lower levels of HDL-cholesterol were the more frequent components in those with MS, and elevated blood pressure was the less frequent.

**Conclusions:**

The high prevalence of MS in the Xavante Indians is mainly due to the increased prevalence of weight excess that resulted from an intense change in their life-style, in a short period of time in a population with a genetic predisposition. These findings highlight the magnitude of this health problem and make an alert about the necessity to implement specific preventive interventions.

## Background

The metabolic syndrome (MS) is defined as a condition where risk factors for cardiovascular diseases and diabetes mellitus (DM) occurs in the same individual. Its main components are central obesity, hypertension, dyslipidemia and abnormalities in the glucose homeostasis [1, 2].

There are few data about the prevalence of MS in the Brazilian Indigenous population. Some reports highlighted the alarming increase of weight excess and of non-communicable diseases in this population, as well changes in their food consumption and reduction in the frequency and intensity of physical activity [3–16]. All these findings suggest that the prevalence of MS could be elevated among Brazilian Indians.

Some studies in specific Brazilian Indigenous communities already produce concerns about the elevated prevalence of MS, that range from 15.5 to 65.3 % [9, 17–20].

The Xavante Indians belong to the Macro-Jê linguistic group and live in a broad region of the Brazilian central plateau, known as “cerrado”, and have a low degree of admixture confirmed by genome-wide analysis [21]. Traditionally hunter-gatherers, and due to conflicts with newcomer farmers, they started to be settled in delimitated areas in a process that started in 1957. This new condition produced important changes in their lifestyle, became more sedentary and modified their traditional diet by incorporating new foods obtained in nearby cities or through food baskets donated by governmental agencies. Thus, important changes have been observed in the nutritional and health profile of this population, including diseases, such as diabetes, that were previously unknown to them [4, 10, 16].

Until now, there was not any information about the frequency of MS in the Xavante population, an Indian group with high prevalence of diabetes and weight excess, two basic conditions in the genesis of this syndrome [22].

Considering that MS has great clinical relevance, and is associated with a higher risk to develop cardiovascular diseases and diabetes [1, 2], it is important to know its prevalence and so its magnitude to plan and to develop specific intervention programs on modifiable risk factors, particularly in the diet and physical activity. The objective of this study was to evaluate the prevalence of MS and the frequency of its components in the adult Xavante population, from the Indian reservations of São Marcos and Sangradouro/Volta Grande, in the state of Mato Grosso.

## Methods and procedures

### Study population

The *Xavante Indians* are estimated to have 20,000 individuals and belong to the Macro-Jê linguistic group, and live in eight Indian reservations in the state of Mato Grosso, Brazil [23–25].

During the field work, the São Marcos and Sangradouro Reservations were visited ten times from October 2010 to January 2012. The total population from these two reservations are estimated in 4065 individuals, being 1582 aged 20 years or more [24, 25].

Local indigenous leaders were previously contacted and provided a written consent for the study, and this was used to get approval from regulatory agencies to carry out the study and to get financial support. Before starting the field work, the local leaders were contacted again, and all the procedures to be performed were explained. All participants agreed to participate and signed an informed consent. For the ones who were illiterate (14 %), fingerprints were used to document their approval, with the aid of a local leader or a Xavante health worker. All examinations and blood sample collections were made in the Indian villages.

All individuals aged 20 years or more were invited to participate in the study. The acceptance of this survey was very high among them, and the non-participants were those absent in the village at the time of the survey. Data were obtained from 932 Xavante Indians from both sexes.

This survey was approved by the Research Ethics Committee (CEP) of the Ribeirão Preto Medical School of the University of São Paulo, by the Brazilian National Ethics Committee (CONEP), and by the Brazilian National Indian Foundation (FUNAI). All procedures were performed in accordance with the ethical principles of the 2008 Declaration of Helsinki.

### Procedures

The variables of interest were: sex, age, weight, height, waist circumference, blood pressure, capillary glycemia (basal and 2-h after 75 g of glucose), serum levels of triglyceride and HDL-cholesterol. Weight was measured using a portable digital scale (Plenna^®^), with a capacity of 150 kg and precision of 0.1 kg, with the individual wearing light clothes and barefoot. Height was measured using a portable stadiometer (AlturExata^®^), with precision of 0.1 cm. Body mass index (BMI) was calculated as the ratio of weight (kg) to the square height (m).

The nutritional status was evaluated according to the proposed WHO criteria [26] for adults: underweight (BMI <18.5 kg/m^2)^; euthrophic (BMI ≥18.5 kg/m^2^ and <25.0 kg/m^2^); overweight (BMI ≥25.0 kg/m^2^ and <30.0 kg/m^2^); obesity (BMI ≥30.0 kg/m^2^). For the elderly were considered the following cutoff points: underweight (BMI ≤22.0 kg/m^2^); eutrophic (BMI >22.0 kg/m^2^ and <27.0 kg/m^2^); Overweight (BMI ≥27.0 kg/m^2^) [27]. For the analysis, the subjects with overweight and obesity were grouped in the category of weight excess.

Waist circumference (WC) was measured at the midpoint between the lowest rib and the upper border of the anterior superior iliac crest, with the individual in the standing position.

Blood pressure was measured in the left arm from seated subjects, after 5 min of rest, using the OMRON HEM-742INTC^®^ equipment. Blood pressure was measured three times and the average of the last two readings was taken as final.

Basal capillary glycemia and the sample taken 2 h after 75 g of anhydrous glucose (Glutol^®^) were measured by a portable glucometer (HemoCue^®^ Glucose 201^+^).

Blood samples were collected from individuals fasting from 8 to 10 h through vein puncture in the forearm using vacuum, sterile, and disposable collectors (Vacuotainer^®^). Blood samples were processed, separated into aliquots, and stored at −20 °C before transportation to the city of São Paulo for laboratory analysis. Serum levels of HDL-cholesterol and triglycerides were measured by enzymatic methods.

The diagnosis of MS was made when the individual have three or more of the following components [2]:

- Basal glycemia > 200 mg/dL or 2-h glycemia > 140 mg/dL or using antidiabetic medication;
- HDL-cholesterol <40 mg/dL for men and <50 mg/dL for women or drug treatment for low HDL;
- Triglycerides ≥150 mg/dL or drug treatment for elevated triglycerides;
- WC ≥94 cm for men and ≥80 cm for women;
- blood pressure >130/85 mmHg or drug treatment for hypertension;

According to the diagnostic criteria for MS, one of the components is diabetes or fasting glycemia ≥100 mg/dL (impaired fasting glucose—IFG). This condition was not considered due to difficulties in assuring the fasting condition. The Xavante Indians do not have a regular schedule for meals and there are cultural barriers to understand the need to fast for the purposes of blood testing. Due to these difficulties, we decided to consider as a proxis of IFG the impaired glucose tolerance—IGT, that is 2-h glycemia in the 140–199 mg/dL range.

In relation to the WC as a component of the MS, there are no specific cut-off points defined for the Brazilian Indian population. The option was to follow the more recent recommendations of the AHA/NHLBI [2], that men with WC ≥94 cm and women with WC ≥80 cm have higher risk to present cardiovascular diseases and diabetes mellitus. Also, these cut-off points are those more frequently adopted in recent publications.

### Statistical analysis

Continuous data were expressed as proportions, means and standard deviations. Means were compared by the Student *t* test and the prevalence rates by the Chi square (χ^2^). Normality of distribution of the variables was tested using skewness/kurtosis test for normality (Kolmogorov–Smirnov test) and all had approximately normal distribution. All analysis were performed using the software statistical package for social sciences (SPSS) version 17. The significance level was fixed at p < 0.05.

## Results

A total of 932 individuals from both sexes (457 men, 475 women) aged 20 years or more, were evaluated. Table 1 presents means and standard deviations of age, clinical, biochemical and anthropometric data, by gender of the study population. The mean age for the study population was 42.7 ± 19.1 years, and was similar for both genders. The overall BMI mean was 30.3 ± 5.1, being slightly higher for women (30.8 ± 5.6 vs. 29.9 ± 4.6; p = 0.007). Women had mean values higher than men for basal and 2-h glycemia, HDL-cholesterol, WC and BMI. Systolic and diastolic blood pressure, weight and height were higher in men. There was no significant difference between genders in triglyceride levels.

**Table 1:** Mean and standard deviation of age, clinical, biochemical and anthropometric data, by gender in the adult Xavante population from São Marcos and Sangradouro/Volta Grande Reservations, 2008–2012

Variables	Mean ± SD			p-value
	Total n = 932	Women n = 475	Men n = 457	
Age (years)	42.7 ± 19.1	42.4 ± 19.3	43.1 ± 19.0	0.556
Basal glycemia (mg/dL)	152.5 ± 105.7	164.1 ± 113.8	140.6 ± 95.2	0.001*
2-h glycemia (mg/dL)	148.7 ± 51.8	158.1 ± 49.0	140.1 ± 52.8	0.001*
HDL-cholesterol (mg/dL)	38.9 ± 8.1	40.7 ± 8.2	37.1 ± 7.5	0.000*
Triglycerides (mg/dL)	199.0 ± 170.9	196.3 ± 179.4	201.8 ± 161.6	0.638
Systolic blood pressure (mm/Hg)	72.7 ± 10.8	71.5 ± 10.6	74.0 ± 10.9	0.000*
Diastolic blood pressure (mm/Hg)	122.3 ± 17.4	119.6 ± 18.5	125.0 ± 15.8	0.000*
Waist circumference (cm)	97.3 ± 10.9	98.6 ± 11.1	95.9 ± 10.4	0.000*
Weight (kg)	78.8 ± 15.3	74.0 ± 14.7	83.8 ± 14.2	0.000*
Height (m)	1.61 ± 0.08	1.55 ± 0.05	1.67 ± 0.05	0.000*
BMI (kg/m^2^)	30.3 ± 5.1	30.8 ± 5.6	29.9 ± 4.6	0.007*

* p < 0.01

Table 2 presents the distribution of the Xavante population, according to nutritional status, by gender and age-group. There was a high prevalence of weight excess, mainly in the age-groups of 20–39 and 40–59 years.

**Table 2:** Distribution of the adult Xavante population, from the Indian Reservations of São Marcos and Sangradouro/Volta Grande, by gender, age-group and nutritional status, 2008–2012

Gender	Nutritional status			
	Underweight	Euthropic	Weight excess	Total
	n (%)	n (%)	n (%)	n (%)
Men				
20–39 years	–	34 (12.9)	229 (87.1)	263 (57.5)
40–59 years	–	10 (9.1)	100 (90.9)	110 (24.1)
≥60 years	7 (8.3)	35 (41.7)	42 (50.0	84 (18.4)
Sub-total	7 (1.5)	79 (17.3)	371 (81.2)	457 (49.1)
Women				
20–39 years	1 (0.4)	24 (8.9)	245 (90.7)	270 (56.8)
40–59 years	–	7 (7.0)	93 (93.0)	100 (21.1)
≥60 years	13 (12.4)	41 (39.0)	51 (48.6)	105 (22.1)
Sub-total	14 (2.9)	72 (15.2)	389 (81.9)	475 (50.9)
General				
20–39 years	1 (0.2)	58 (10.9)	474 (88.9)	533 (57.2)
40–59 years	–	17 (8.1)	193 (91.9)	210 (22.5)
≥60 years	20 (10.6)	76 (40.2)	93 (49.2)	189 (20.3)
Total	21 (2.3)	151 (16.2)	760 (81.5)	932 (100.0)

**Fig. 1:**
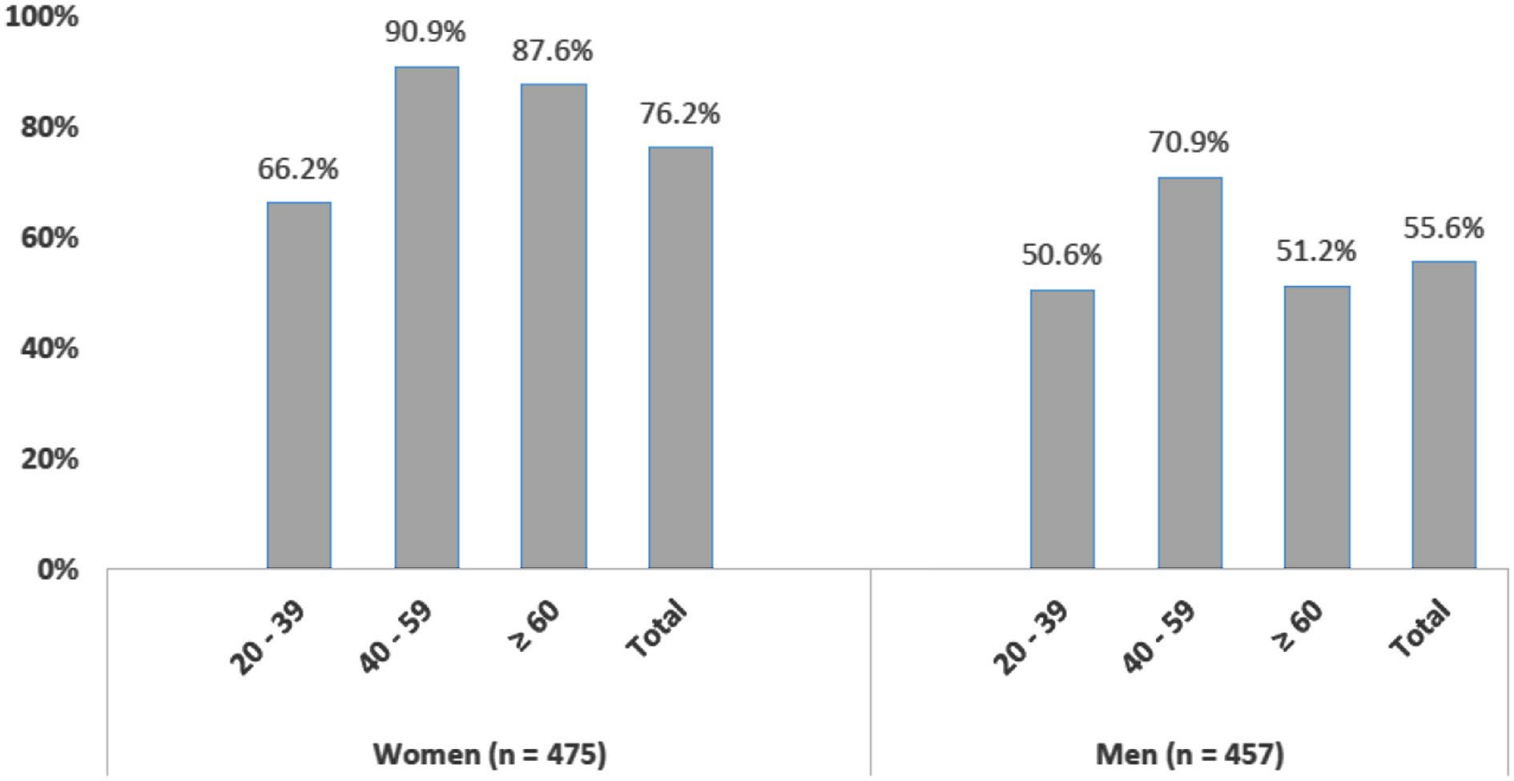
Prevalence of metabolic syndrome by gender and age-group in the adult Xavante population of the São Marcos and Sangradouro/Volta Grande Reservations, 2008–2012

It was observed that 66.1 % (95 % CI 63.0–69.2) of the Xavante Indians presented MS, being 76.2 % (95 % CI 72.4–80.0) in women and 55.6 % (95 % CI 51.0–60.2) in men, and the difference was significant (p < 0.001). The prevalence rates of MS were higher in women than in men, in all age-groups (Fig. 1).

Among women, 23.8 % presented less than three components of the MS and all presented at least one of its components. Among men, the frequency of less than three components of the MS was 34.0 and 2.2 % (20 individuals) did not have at least one of its component (Table 3).

**Table 3:** Frequency of the components of metabolic syndrome, by gender, in the adult Xavante population from São Marcos and Sangradouro/Volta Grande Reservations, 2008–2012

Components of metabolic syndrome	Women n (%)	Men n (%)	Total n (%)
None	– (–)	20 (4.4)	20 (2.2)
1	24 (5.1)	77 (16.9)	101 (10.9)
2	88 (18.7)	105 (26.1)	193 (20.9)
3	143 (30.4)	139 (30.5)	282 (30.5)
4	151 (32.1)	88 (19.3)	239 (25.8)
5	64 (13.6)	26 (5.7)	90 (9.7)

Elevated WC and lower levels of HDL-cholesterol were the most frequent components of the MS, and elevated blood pressure was the less frequent component (Table 4).

**Table 4:** Frequency of the components in those with metabolic syndrome, by gender and age-groups, in the adult Xavante population from São Marcos and Sangradouro/Volta Grande Reservations, 2008–2012

Age-groups (years)	Components of metabolic syndrome (%)				
	Hypertension n (%)	Low HDL-cholesterol n (%)	Elevated triglycerides n (%)	Hyper-glycemia n (%)	Elevated waist circumference n (%)
Women					
20–39	32 (18.2)	164 (93.2)	103 (58.5)	147 (83.5)	175 (99.4)
40–59	40 (44.4)	85 (94.4)	68 (75.6)	84 (93.3)	90 (100.0)
≥60	49 (53.3)	79 (85.9)	67 (72.8)	82 (89.1)	88 (95.7)
Sub-total	121 (33.8)	328 (91.6)	238 (66.5)	313 (87.4)	353 (98.6)
Men					
20–39	56 (42.4)	110 (83.3)	108 (81.8)	73 (55.3)	113 (85.6)
40–59	41 (52.6)	60 (76.9)	63 (80.8)	53 (67.9)	67 (85.9)
≥60	35 (81.4)	31 (72.1)	28 (65.1)	28 (65.1)	33 (76.7)
Sub-total	132 (52.5)	201 (79.4)	199 (78.7)	154 (60.9)	213 (84.2)
Total	253 (41.4)	529 (86.6)	437 (71.5)	467 (76.4)	566 (92.6)

Among those with MS, the frequency of high blood pressure and elevated triglycerides were more frequent among men, whereas the other components were more frequent among women (Table 4).

## Discussion

It was observed, in the last few decades, a process of approximation between the Xavante population and the urban communities of nearby cities of the Xavante Reservations, which induced modifications in the relationship of the Indians with the land, work and their way of getting foods.

Factors as territorial restrictions, exhaustion of natural resources, alterations in their pattern of settlement and loss of their nomadic life resulted in a more sedentary lifestyle of these Indians. They also incorporated several foods from the Brazilian society and started to have industrialized foods, rich in sugar, fat and salt, and poor in proteins, fibers and several micronutrients [4, 7, 10, 16].

All these changes contributed to modify the epidemiologic profile of these populations, with the increase in the prevalence of non-communicable diseases, as obesity, hypertension, type 2 diabetes and metabolic syndrome.

The results from the present study showed an elevated prevalence (66.1 %) of MS among the adult Xavante Indians, indicating a high risk for cardiovascular diseases and type 2 diabetes. This prevalence rate is higher than the most reported ones for Brazilians, either for indigenous populations [17–20] or non-indigenous [28].

A systematic review about the prevalence of MS in Brazilian adults, selected ten studies, from which two were performed in indigenous populations, and the average prevalence was 29.6 % (14.9–65.3 %). The highest rate was observed among the Kaingang and Guarani (65.3 %), from the state of Rio Grande do Sul, being very similar to results from the present study [28].

Other studies with Indian populations found lower prevalence of MS, as 15.5 % in the Karib Indians from Upper Xingu [17], 21.9 % among the Indian Suya [9] and 27.8 % among the Khisêdjê [20], groups from the Xingu Indian Park, in the state of Mato Grosso; 23.3 % among the Kaingang Indians from the state of Rio Grande do Sul [18] and 35.7 % among Indians from the village of Jaguapiru, in the municipality of Dourados, Mato Grosso do Sul [19].

Comparing the prevalence of MS from the present study with data published from other countries, it is possible to realize that the Xavante rate for MS is very superior than the reported rates for Portugal (27.6 %) [29], Spain (26.6 %) [30], France (25 % in men and 15.3 % in women) [31], Italy (28 % in men and 26 % in women) [32], United States of America (22.9 %) [33], Mexico (26.6 %) [34], Peru (18.1 %) [35], Chile (29.5 %) [36], North of Africa (30 %) [37], Colombia (34.8 %) [38], Venezuela (35.3 %) [39], China (33.9 %) [40] and Turkey (36.6 %) [41].

The same was observed when the rate from the Xavante population was compared with prevalence rates from indigenous populations from other countries, as 10 % in Siberia [42], 38 % in Argentina [43] and 46.9 % in Austrália [44].

There are large differences in the prevalence of MS, according to the study population and the place of the study. Part of the differences are due to different diagnostic criteria to define the components of the MS, particularly the WC. The definition of the cut-off points for the WC, an important component to evaluate the central obesity, varies according the ethnicity, which limits this parameter in a multiethnic population.

The higher rates found among Xavante women are similar to the findings reported from other studies [9, 17–20, 28]. These findings raises the hypothesis that other variables, not yet identified, may affect the association between gender and presence of MS in the Indian population.

The Xavante Indians from the age-group 40–59 years were those with the higher rates of MS in relation to the younger and older age-groups. In a systematic review [28] about the prevalence of MS in Brazilian adults, it was described that the prevalence of MS increased with the age, being more elevated in individuals aged 50 years or more.

The fact that the Xavante Indians aged 60 years or more did not present the highest rates for MS can be explained by a less intense process of acculturation of the elderly, greater maintenance of their traditional habits and less contact with the Brazilian society due to language barriers, resulting in lower changes in their life style, particularly in the diet and physical activity.

Lower HDL-cholesterol levels were very frequent in other Brazilian studies with indigenous [17–20] and non-indigenous [28] populations. In the Xavante population, besides their genetic background, could also contribute to the lower HDL-cholesterol levels, the reduction in their physical activities and a low consumption of nuts and monounsaturated fats.

The frequency of elevated WC was more frequent in the Xavante population than that reported in other Brazilian Indian populations [17–20, 28]. Presently, there are no specific WC cut-off points proposed for the diagnosis of MS in the Indigenous population. Because of this lack of definition, several values are used. There is a need to define specific cut-off points for the WC for the Indian populations, that have differentiated structure and body composition.

Although relevant, high blood pressure was the less frequent component of the MS in the Xavante. In a recent study [22], the age-adjusted prevalence of hypertension in this population was 17.5 %, which is below the average values reported for the general adult Brazilian population [45]. Results from the First National Health and Nutritional Survey in indigenous people [3] showed that 13.1 % of the women aged 14–49 years presented high blood pressure. A systematic review about MS in the general adult Brazilian population, showed that 52.5 % of the individuals had abnormal values for blood pressure [28].

Abnormalities in the glucose homeostasis are an important health problem among the Xavante population. A recent survey [22] showed that the age-adjusted prevalence rates for diabetes and impaired glucose tolerance were 28.2 and 32.3 %, respectively.

The definition of a single cause or of multiple causes for the development of MS are still a challenge, but central obesity and insulin resistance have a key role in its Genesis. With the hypertrophy of the adipose tissue, occurs an unbalance in the secretion of pro and anti-inflammatory adipokines, including the tumor necrosis factor alpha (TNF-α), the interleukin-6 (IL-6) and adiponectin, establishing a state of chronic inflammation of low intensity, that represents an important role in the development of obesity-related comorbidities. Concomitantly occurs an activation of pathways of inflammatory signals, as c-Jun N-terminal kinase (JNK) and transcription nuclear factor κB (NF-κB), that produces insulin resistance [46–49].

The high prevalence rate of MS in the Xavante Indians create a high risk for the development of cardiovascular diseases and type 2 diabetes. It is known that the presence of MS increases twofolds the risk for cardiovascular diseases and of fivefolds for type 2 diabetes [2, 50, 51].

In spite that these results are not representative of all Brazilian Indian populations, they are very important for the Xavante, since the size of our study sample corresponds to approximately 60 % of the adult population of the São Marcos and Sagradouro/Volta Grande reservations. Our findings highlighted the importance of this health problem among the Xavante Indians and calls for the need for preventive measures towards risk factors for cardiovascular diseases.

## Conclusions

The high prevalence of MS among the Xavante Indians, besides the genetic predisposition, is related to the high frequency of weight excess, due to changes in the pattern of food consumption and reduction in the frequency and intensity of physical activity. These results make an alert about the magnitude of this health problem, that will increase in a near future and resulting in a great impact in the quality of life of these Indians. There is an urgent need to have specific interventions towards prevention of risk factors for cardiovascular diseases.

## Abbreviations

MS: metabolic syndrome
DM: diabetes mellitus
BMI: body mass index
WC: waist circumference
IFG: impaired fasting glucose
IGT: impaired glucose tolerance
CONEP: Brazilian National Ethics Committee
FUNAI: Brazilian National Indian Foundation
WHO: World Health Organization
TNF-α: tumor necrosis factor alpha
IL-6: interleukin-6
JNK: c-Jun N-terminal kinase
NF-κB: transcription nuclear factor κB
